# Morpho‐Physiological, Root Exudation and Metabolomics Insights on the Single and Combined Effect of Exogenous Strigolactones and Mycorrhiza in Tomato

**DOI:** 10.1111/ppl.70814

**Published:** 2026-02-20

**Authors:** Biancamaria Senizza, Leilei Zhang, Begona Miras‐Moreno, Pascual Garcia‐Perez, Paolo Bonini, Fabrizio Araniti, Luigi Lucini

**Affiliations:** ^1^ Department for Sustainable Food Process Università Cattolica del Sacro Cuore Piacenza Italy; ^2^ Department of Plant Biology, Faculty of Biology University of Murcia, Campus de Espinardo Murcia Spain; ^3^ Department of Food Technology, Nutrition and Food Science, Veterinary Faculty University of Murcia, Regional Campus of International Excellence “Campus Mare Nostrum” Murcia Spain; ^4^ oloBion‐OMICS LIFE LAB Barcelona Spain; ^5^ Department of Agricultural and Environmental Sciences ‐ Production, Landscape, Agroenergy University of Milan Milan Italy

**Keywords:** abiotic stress, GR‐24, phytohormones, plant signaling

## Abstract

Strigolactones (SLs) are plant hormones and have recently been characterized for their regulatory roles in plant development and rhizosphere communication, including interactions with arbuscular mycorrhizal fungi (AMF). The present work investigated the single and combined effects of exogenous GR24, an SL analogue, and AMF in tomato plants by assessing their potential synergistic effects on morpho‐physiological enhancements and on AMF colonization through root‐untargeted metabolomics and root exudate profiling. Specifically, a supervised multi‐omics data integration approach has been used to identify the most relevant features correlated with treatments. Although the effect of AMF appeared to be hierarchically prevalent, GR24 provided significant effects at all levels when applied alone or combined with AMF. Noteworthy, the combination of GR24 and AMF delivered synergistic effects on morphological and photosynthetic traits with reduced energy loss in photosystem II (PSII) and increased chlorophyll content, and provided distinctive metabolic profiles compared to the single treatments. Mostly in the presence of the GR24 + AMF treatment, a positive modulation of N‐containing compounds, phenylpropanoids, terpenes, and phytoalexins and an adjustment of phytohormones like abscisic acid, brassinosteroid, cytokinin, and jasmonate crosstalk was observed. Moreover, the exudation signatures were consistently distinctively shaped, enriched in flavonoids and amino acids. Finally, the multi‐omics data integration underlined a positive correlation between the roots and root exudate datasets, with high discriminatory potential of the selected features among the treatments.

## 1 Introduction

Strigolactones (SLs) are a class of terpenoid lactones, recently classified as phytohormones, possessing dual functions. These molecules, together with participating in the regulation of plant growth and development, act as signaling molecules in the symbiosis with arbuscular mycorrhizal fungi (AMF) through the promotion of hyphal branching and the activation of the fungal metabolism (Yoneyama and Brewer 2021). AMF are obligate biotrophs that colonize the root cortex and exchange nutrients with the plant by developing arbuscules in the host's cells (Andreo‐Jimenez et al. 2015). During this mutualistic symbiosis, the plant provides carbohydrates and lipids to the fungus, which in turn provides photosynthetic minerals, mainly phosphorus and nitrogen, to the plant. Under phosphate deficiency, plants produce and exude SLs to repress branching, increase the root area, and promote this symbiosis, thereby supporting the acquisition of inorganic nutrients (Yoneyama 2019). Evidence indicates that the synthetic analogue of SL, called GR24, acts as a germination prompt for AMF spores and as a significant communication signal that modulates root architecture and symbiotic efficiency (Liao et al. 2018; Boyno et al. 2023). This signaling pathway highlights the central role of GR24‐AMF interactions in shaping plant development and strengthening resilience to abiotic stresses. Recent studies have emphasized that the GR24‐AMF axis not only enhances nutrient uptake, modulates hormonal networks, and improves plant tolerance to challenging environmental conditions (Faizan et al. 2020; Demir et al. 2022), but also facilitates the initiation of AMF colonization (Boyno et al. 2023).

The efficacy of the symbiosis differs depending on the species of the fungi and the plant species. Still, this mutualism has a positive impact on the plant's development, especially under adverse growing conditions. In tomato plants, this interplay between GR24 and AMF demonstrated the ability to fine‐tune root‐soil signaling networks, facilitating more efficient phosphorus and micronutrient acquisition while potentially reprogramming root system architecture to optimize resource allocation under stress conditions (Bona et al. 2017).

Aside from their role as signalling molecules, SLs inhibit shoot branching, reduce lateral root growth, and control the delay in leaf senescence and secondary root growth (Chen et al. 2022). These phytohormones are active at low concentrations, from pico‐ to nanomolar and each plant can generate different types and quantities of SLs depending on various types of abiotic stresses and growth conditions (Mostofa et al. 2018). In response to abiotic stresses, SLs also interact with other phytohormones such as abscisic acid, cytokinins, and gibberellins, underlining their contribution to the regulatory system of plant stress adaptations (Bhoi et al. 2021). When applied exogenously, SLs enhance tolerance to drought, to heat and to osmotic stresses by adjusting the relative water content, antioxidant enzyme activities, and membrane stability index (Sedaghat et al. 2017; Omoarelojie et al. 2020; Li et al. 2020). Under salinity conditions, the symbiosis with AMF controls the production of SLs to cope with the stress (Chen et al. 2022). Concurrently, AMF play a proven role in enhancing plant growth and productivity through multiple physiological and biochemical mechanisms, especially in tomato plants ( *Solanum lycopersicum* L.), where their effects on nutrient uptake, hormonal regulation, and stress tolerance have been extensively characterized (Bona et al. 2017; Singh et al. 2024). This symbiotic association boosts photosynthetic efficiency, root development, and biomass accumulation (Shafiq et al. 2023). Moreover, AMF have been shown to elevate the concentration of bioactive compounds such as lycopene and flavonoids, thereby improving fruit quality (Singh et al. 2024). Under abiotic stress conditions, such as drought, AMF contribute to water regulation and osmotic balance, enhancing plant resilience (Leventis et al. 2021). These multifaceted benefits underscore the potential of AMF as a sustainable biofertilizer in tomato cultivation systems (Demir et al. 2022). In the context of hormonal signaling, AMF colonization modulates plant hormonal landscapes, including auxin, cytokinin, jasmonate, and salicylic acid pathways (Umer et al. 2025), thereby creating a feed‐forward loop in which GR24‐mediated and AMF‐derived signals converge to optimize stress‐responsive gene networks and metabolic adjustments. Nevertheless, no studies have demonstrated that GR24‐AMF can be combined with eliciting plant growth and performance in a synergistic manner, without observing plant growth penalties under favorable conditions, particularly at early‐stage colonization by AMF.

This work investigated the complex interplay between SLs and AMF in tomato plants ( *Solanum lycopersicum* L.). For this purpose, a comprehensive multi‐omics strategy was employed, combining untargeted metabolomic profiling of roots and root exudates with detailed morphological assessments. By integrating these datasets, the research aimed to better understand the dynamic interactions among root metabolism, exudate profiles, and AMF under both GR24‐treated and untreated conditions. Additionally, morphological and physiological parameters were examined to provide insights into overall plant health and the systemic impact of SL‐AMF signaling.

## 2 Materials and Methods

### 2.1 Plant Material, Growth Conditions, and Experimental Design

Tomato plants ( *Solanum lycopersicum* L., cv. Heinz 3402) were grown for 35 days, starting from 10th June 2021 to 22nd July 2021, under natural open field conditions at the experimental station of Università Cattolica del Sacro Cuore (Piacenza, Italy). The thermometric parameters are reported in the Supporting Information.

Tomato seedlings were provided by a local nursery (Azienda F.lli Zermani, Piacenza, Italy) and transplanted in single round pots (15 cm × 12.5 cm) at the four true leaf stage. The soil substrate was commercially made of neutral fine peat (Vigorplant Italia Srl, Fombio, LO, Italy). The specific characteristics of the soil include a 7.5 pH value in water, 0.4 dS m^−1^ electrical conductivity, 180 kg m^−3^ density, and 87% v v^−1^ total porosity.

The experimental design is composed of: control untreated, 15 mM of strigolactone (GR24; Symeres Netherlands B.V.), commercially available AMF inoculum *(*25 spores g^−1^ *of Rhizoglomus irregulare* BEG72 and 25 spores g^−1^ of *Funneliformis mosseae* BEG234—Aegis Sym irriga), and 15 mM GR24 + AMF. Specifically, the AMF inoculation was applied after 1 week of adaptation post‐transplantation. AMF was applied according to label recommendations: a single application at 0.1 g per plant at transplanting. GR24 was applied 5 mL × 8 times by foliar spray: 7, 8, 9, 14, 21, 28, 35, and 42 days after transplant (DAT). The concentration of GR24 (15 mM) was chosen based on literature reviews, reporting a range between 1 and 15 mM (Lu et al. 2019; Li et al. 2024). The experiments were arranged in a completely randomized design, with seven biological replicates for each treatment, amounting to 28 pots in a single experiment.

At the end of the experiment, plants were harvested and divided into two groups: four out of seven replicates were used for morphological analysis and root exudate collection, and the remaining plants were used for metabolomics and AMF root colonization assays. Specifically, the roots of tomato plants reserved for morphological analysis were washed under tap water and then incubated in 200 mL of Milli‐Q water for 4 h to collect the root exudates. Then, 40 mL of root exudates were centrifuged at 6000 × g for 20 min, filtered through a 0.22 mm membrane, and freeze‐dried for analysis. At the same time, the shoots were separated from the roots, and their fresh (FW) and dry weight (DW) were determined before root morphology characterization. Concerning metabolomics, roots were gently washed with tap water, snap frozen in liquid nitrogen, and immediately stored for metabolomics analysis.

### 2.2 Root System Characterization

The characterization of the root system was executed as previously described by Lupini et al., with some variations (Lupini et al. 2014). The tomato roots were collected, cleaned from soil residues under tap water, and maintained in a solution composed of ethanol (96%; 656.25 mL l^−1^), formaldehyde (37%; 50 mL l^−1^) and glacial acetic acid (99.85%; 50 mL l^−1^). Before analysis, the roots were stained for 5–10 min (depending on the diameter of the roots) in a 0.1% Toluidine blue solution and repeatedly washed in deionized water to eliminate the dye in excess. Following these steps, because of the dimensions of the apparatus, the roots were separated into portions and placed onto a waterproof tray filled with distilled water and scanned using an Epson scanner (Perfection V850 pro). The images were analysed using RhizoVision Explorer v2.0.3 (Seethepalli et al. 2021) using the algorithms described by Seethepalli et al. (2021). The parameters obtained were total root lengths, projected area (ProjArea), surface area (SurfArea), root volume (RootVolume), average diameter (AvgDiam), root length ratio (RLR), root mass ratio (RMR), root fineness (RF), and root tissue density (RTD).

### 2.3 Photosynthetic Activity Measurements

The MultispeQ 2.0 device (PhotosynQ) was used for photosynthetic analysis of the treated tomato plants. Five plants per treatment were randomly chosen for data acquisition. The analyzed parameters included the quantum yield of photochemical energy conversion in PSII (Phi2), the quantum yield of non‐regulated non‐photochemical energy loss in PSII (PhiNO), and the quantum yield of regulated non‐photochemical energy loss in PSII (PhiNPQ), which were calculated by applying the equations derived by Kramer et al. (2004) as modified by Tietz et al. (2017). Accordingly, the three light‐adapted parameters add up to 1 (Phi2 + PhiNPQ + PhiNO = 1).

### 2.4 Root and Root Exudate Metabolomics Analysis

Root tissues and root exudates were analyzed using a 1290 UHPLC chromatograph coupled to a QTOF mass analyzer (G6550 mass spectrometer, Agilent Technologies) equipped with an electrospray ionization (ESI) source as described in the Supporting Information. Specifically, 1 g of root tissues was dissolved in 10 mL of 80% methanol + 0.1% formic acid and homogenized for 3 min (Polytron PT 1200 E, Kinematica AG). For root exudates, the freeze‐dried matters were resolubilized on 1 mL of ultrapure water (Carlo Erba, Cornaredo). Both extracts were centrifuged at 5000 × g for 15 min and filtered (0.22 μm membrane) into vials for UHPLC analysis. The chromatographic separation and QTOF mass spectrometer analysis are reported in detail as Supporting Information (Table S2).

### 2.5 Arbuscular Mycorrhiza Fungi (AMF) Root Colonization

The three replicates of each experimental group were subjected to AMF root colonization quantification. Specifically, root samples of tomato plants were rinsed, and subsamples were used to evaluate AMF root colonization. The root samples were cleared with 10% (w/v) KOH, stained with 0.05% (w/v) trypan blue in lactophenol, and microscopically examined for AMF colonization (Stereo microscope Leica EZ4V, 32×—Leica Microsystems Srl). The percentage of colonized root segments was determined by the grid line to intersect the method (Bonini et al. 2020).

### 2.6 Data Integration

The multi‐omics data integration analysis to correlate the roots and exudate datasets was achieved through the mixOmics package from the R statistical program (version 4.1.3, R Core Team 2022) using the DIABLO framework (Data Integration Analysis for Biomarker Discovery using Latent Variable Approaches for Omics Studies), a multi‐omics classification and integration method. The assessment of the correlation structure was carried out at the component level for each treatment. The optimal number of principal components and selected variables for the analysis were determined using the tuning function. The number of components was chosen based on the balanced error rate (BER) that reached the best performance. Conversely, the number of variables was established through repeated and stratified cross‐validation to compare the model performance constructed employing different ℓ1 penalties.

### 2.7 Statistical Analysis

One‐way analysis of variance (ANOVA), followed by the Duncan multiple range test (*p* < 0.05), was conducted on different physiological measurements by using the software PASW Statistics 26.0 (SPSS Inc.).

Concerning metabolomics, the software Agilent Mass Profiler Professional B.15.1 (Agilent Technologies) was used for data filtration (area threshold > 10,000 counts), Log2‐transformation and normalization at the 75th percentile as previously described (Sorrentino et al. 2021). The detected features were baselined to their median in the dataset. Following that, an unsupervised hierarchical cluster analysis (HCA) was conducted using a fold‐change heat map (Euclidean similarity measure and ‘Wards’ as linkage rule). Normalized resulting data were Pareto‐scaled for multivariate data analysis by SIMCA version 17 software (Sartorius).

Differential metabolites were identified by Volcano Plot analysis, combining ANOVA and fold‐change analysis: statistically significant compounds (*p* < 0.05, Bonferroni multiple testing correction) and log Fold Change ≥ 1.2 were considered for the pathway analysis using the Omic Viewer Pathway Tool of PlantCyc (Stanford) for biochemical interpretations (Caspi et al. 2013). Subsequently, Chemical Similarity Enrichment Analysis (ChemRICH) was performed using an online tool available at http://chemrich.fiehnlab.ucdavis.edu, considering only the differential compounds (Barupal and Fiehn 2017). The selected discriminant compounds were those displaying significantly (*p* < 0.05) different abundances and logarithms of fold‐change (logFC) values > 1.2.

## 3 Results

### 3.1 Morphological and Physiological Analysis of Tomato Plants

**FIGURE 1:**
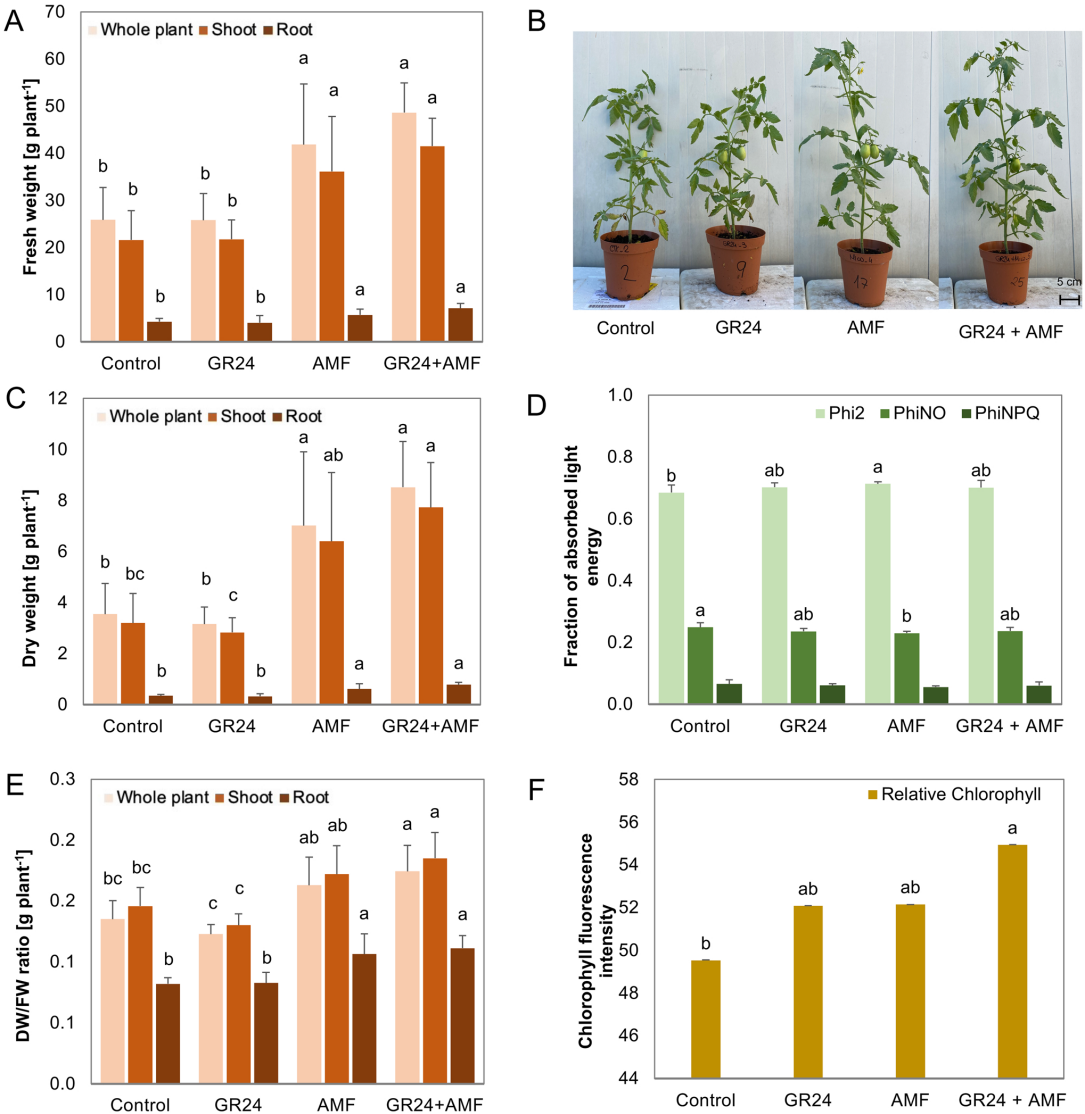
Morphological and physiological parameters of tomato plants in the control and treatment with 15 mM of strigolactones (GR24), arbuscular mycorrhizal fungi (AMF), and their combination (GR24 + AMF). (A) Fresh weight (FW); (B) Pictures of tomato plants at the end of the experiment; (C) Dry weight (DW); (D) Photosynthetic parameters evaluated as the quantum yield of photochemical energy conversion in PSII (Phi2), the quantum yield of non‐regulated non‐photochemical energy loss in PSII (PhiNO), and the quantum yield of regulated non‐photochemical energy loss in PSII (PhiNPQ). (E) DW/FW ratio. (F) Relative chlorophyll content. The bar plots are expressed as mean ± standard deviation. According to Duncan's multiple range test, different letters indicate statistically significant differences within each plot (*p* = 0.05). No letter indicates non‐statistically significant.

Morpho‐physiological parameters, including biomass of root and shoot fresh weight (FW), dry weight (DW), and DW/FW ratio, are reported in Figure 1A,C,E, respectively. Photosynthetic parameters were also determined in terms of the quantum yield of photochemical energy conversion in PSII (Phi2), PhiNO, and the quantum yield of regulated non‐photochemical energy loss in PSII (PhiNPQ), as reported in Figure 1D, together with relative chlorophyll content (Figure 1F).

Plants inoculated with AMF and GR24 + AMF showed improved growth and development, detecting a statistically significant increase in shoot, root, and overall total plant FW and DW compared to the control (Figure 1A,C). An exception was observed for AMF treatment in DW, where shoot growth was not significantly increased compared to the control, suggesting a synergistic effect of the AMF + GR24 combination (Figure 1C). This synergistic effect was also confirmed by considering the DW/FW ratio, reporting a significantly increased value under the GR24 + AMF treatment (Figure 1E). The latest observation suggests the effect of the combined treatment on shoot biomass and the number of tomato branches (Figure 1B). Interestingly, a synergistic effect has been observed in the interaction with GR24 and AMF, highlighting significant shoot development when DW was considered (Figure 1C).

The treatments also affected photosynthetic performance, quantified as the photochemical energy conversion quantum yield in PSII. The non‐regulated non‐photochemical energy loss in PSII decreased, suggesting general plant health. Although no synergistic effect on the photosynthetic performance could be observed, relevant increased chlorophyll content was found under the combined GR24 + AMF application, strengthening the hypothesis of synergistic effects.

### 3.2 Root AMF Colonization

Root AMF colonization was evaluated to explore the impact of various treatments on the extent of mycorrhizal colonization and propagation (Figure S1). The findings revealed an overall 25% AMF root colonization in plants exposed to AMF only. Co‐exposure with GR24 demonstrated a similar degree compared to plants exposed to AMF only.

### 3.3 Root Morphology

Root morphology was determined in terms of total root lengths, projected area (ProjArea), surface area (SurfArea), root volume (RootVolume), average diameter (AvgDiam), root length ratio (RLR), root mass ratio (RMR), root fineness (RF), and root tissue density (RTD). All values are reported in Table S1.

**FIGURE 2:**
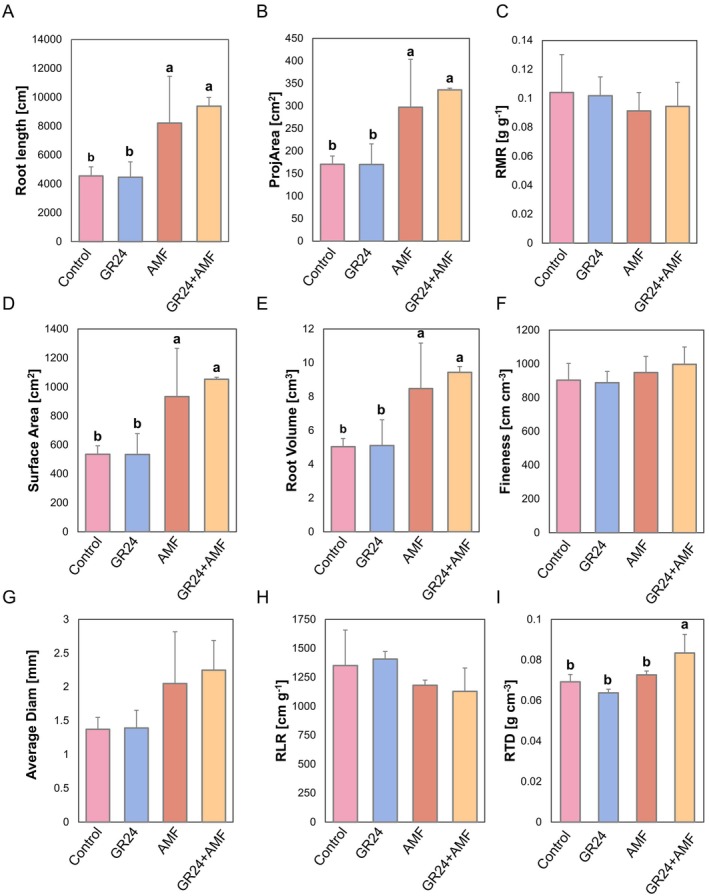
Root morphological parameters of tomato plants in control and treated with 15 mM of the strigolactone GR24, arbuscular mycorrhiza fungi (AMF), and their combination (GR24 + AMF). (A) root length. (B) projected area (ProjArea); (C) root mass ratio (RMR); (D) surface area (SurfArea); (E) root volume; (F) fineness; (G) average diameter (AvgDiam); (H) root length ratio (RLR); (I) root tissue density (RTD). The bar plots are expressed as mean ± standard deviation. According to Duncan's multiple range test, different letters indicate statistically significant differences within each plot (*p* = 0.05). No letter indicates non‐statistically significant.

As expected, AMF significantly increased root length (Figure 2A), root projected area (Figure 2B), root surface area (Figure 2D), and root volume (Figure 2E). Notably, when combined with GR24, the values increased markedly compared to GR24 alone and the untreated controls. Only the combined treatment GR24 + AMF significantly improved root tissue density, whereas no significant variation (*p* < 0.05) was pointed out for the single treatment. Regarding the root mass ratio (RMR), fineness (RF), and the average diameter and length ratio (RLR), there were no significant (*p* < 0.05) differences between treatments.

### 3.4 Root Metabolomics

The effect of GR24, AMF, and their interactions on tomato plants was also evaluated at the metabolome level. Our untargeted approach enabled us to putatively annotate more than 3000 compounds (Table S2), which was further used to elucidate the biological processes implied in plant response to treatments. Firstly, based on the fold change and normalization by median, the unsupervised HCA (Figure S2) was constructed to naively inspect the relatedness of tomato root metabolomes according to the three different experimental conditions considered. The heatmap outlined a hierarchically lower effect of GR24, due to a metabolic similarity between control and GR24, while combined GR24 + AMF and AMF alone clustered apart. The following PCA (Figure S3) confirmed this output, with differences between the control and the GR24 + AMF on component 1, which explains the 14.39% variation. These unsupervised analyses suggest a hierarchically prevalent effect of AMF, even though the effect of GR24 was still evident.

**FIGURE 3:**
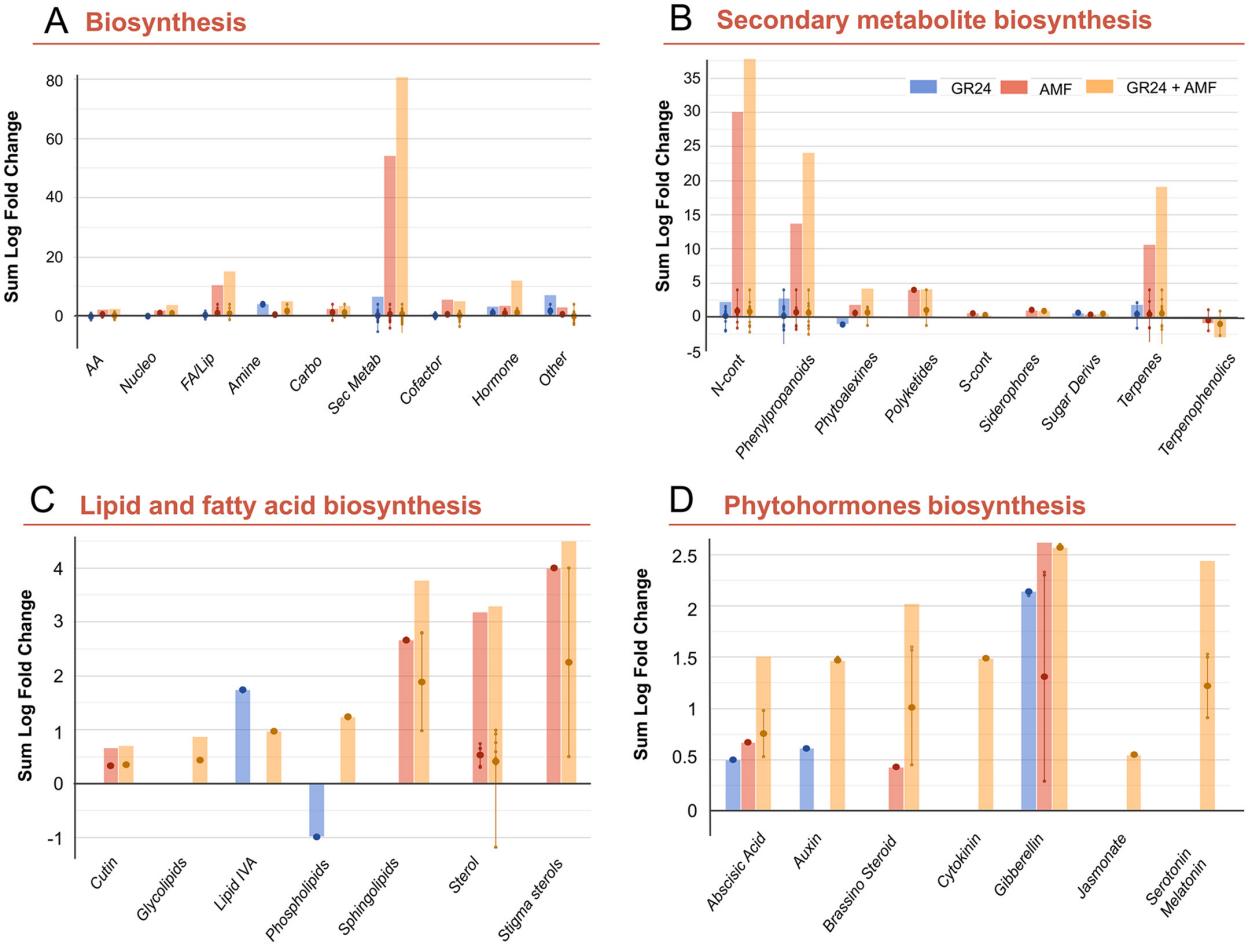
Pathway analysis of tomato plants in control and treated with 15 mM of the strigolactone GR24, arbuscular mycorrhiza fungi (AMF), and their combination (GR24 + AMF). (A) root biosynthetic processes, (B) specialised metabolite biosynthesis, (C) fatty acids and lipids, and (D) hormones. The metabolomics dataset was subjected to ANOVA and log fold change (log FC) analysis (*p* < 0.05, FC ≥ 2). Differential metabolites were loaded into the PlantCyc Pathway Tool (https://www.plantcyc.org/). The bar plots represent the sum of log FC values of metabolites belonging to a specific metabolite class, calculated from pairwise comparisons between treatments and the control. The dot indicates the median of the log FC regulation for the metabolite class. AA, amino acids; Nucleo, nucleotides; FA/Lip, fatty acids and lipids; Carbo, carbohydrates; Sec Metab, secondary metabolites; N‐cont, N‐containing compounds; S‐cont, S‐containing compounds; and Sugar Deivs, sugar derivatives.

Thereafter, Volcano Plot analysis (*p* < 0.05 with Bonferroni multiple testing correction; FC > 1.2) was performed to identify the differential compounds for pathway analysis (PlantCyc) interpretations (Figure 3).

Looking at the results, the treatments induced an impact mainly involving secondary metabolites, fatty acids, and hormones (Figure 3A). Overall, positive modulation of N‐containing compounds, phenylpropanoids, terpenes, and, to a lesser extent, phytoalexins (Figure 3B) was noted, mostly in the presence of the AMF and in combination with strigolactones (GR24 + AMF), outlining their synergistic effect again. Going into detail, GR24 seems to not induce the accumulation of lipids, except for phospholipids, with a general decrease of these compounds (Figure 3C). Conversely, an accumulation of sphingolipids, sterols, and phospholipids was observed in the presence of GR24 + AMF and with the AMF (Figure 3C). Similarly, GR24 + AMF induced a positive impact on the phytohormone profile with a general increase of abscisic acid, auxins, brassinosteroids, and cytokinins (Figure 3D). The combined treatment was also able to determine an increase in jasmonates, not outlined in the other two treatment conditions (Figure 3D). Moreover, the AMF and SLs seemed to strongly affect the modulation of gibberellins and abscisic acids. Interestingly, the first (AMF) could accumulate auxins, and the second (GR24) triggered brassinosteroid accumulation.

### 3.5 Effect on Root Exudation Patterns

**FIGURE 4:**
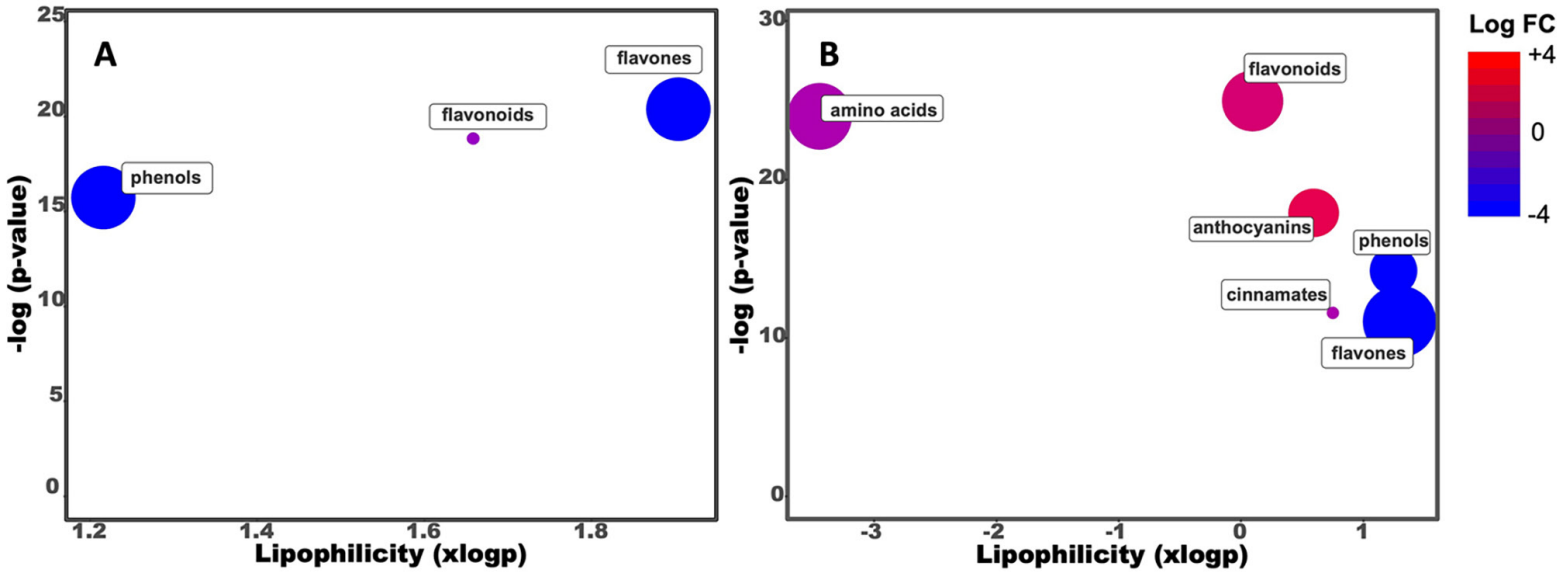
ChemRICH analysis of tomato root exudates treated with (A) AMF and (B) GR24 + AMF. Each node reflects a significantly altered cluster of metabolites. Enrichment *p*‐values are given by the Kolmogorov–Smirnov test. Node sizes represent the total number of metabolites in each cluster set. The node color scale shows the proportion of increased (red) or decreased (blue) compounds. Purple‐color nodes have both increased and decreased metabolites. The y‐axis shows the most significantly altered clusters at the top.

Following the evaluation of the effect of GR24, AMF and their interactions at the metabolome level, the profiling of root exudates through UHPLC‐QTOF‐MS was also performed (Figure 4). This approach allowed us to annotate 471 metabolites comprising isobars (Table S3). The chemical similarity enrichment analysis (ChemRICH) was employed to assess the comprehensive impact of AMF, SLs, and their combination on root exudate signatures and identify the compounds influenced mainly by the treatments. Specifically, this analysis is based on metabolites that passed ANOVA and fold‐change thresholds. For the ANOVA analysis, each treatment was compared with the control, and the number of reported metabolites was 11 (GR24 vs. control), 52 (AMF vs. control), and 80 (AMF + GR24 vs. control). The ChemRICH analysis reported no significant differences between GR24 and the control at the exudate profile level. While the outcome of the enrichment plots following AMF application suggested a general decrease of the phenols and flavones clusters, with a significant decrease of flavonoids, except for dihydroxy kaempferol, which increased (Figure 4A).

Similarly, these classes of compounds were reduced following the combined GR24 + AMF treatment (Figure 4B), while the flavonoids, mainly dihydroxy kaempferol, hesperidin, and isorhamnetin 3‐*O*‐glucoside 7‐*O*‐rhamnoside, were enhanced. An improved anthocyanin content was observed, including cyanidin‐3‐*O*‐(6″‐malonyl‐glucoside), cyanidin‐3‐*O*‐arabinoside, and petunidin‐3‐*O*‐rutinoside, while a general increase/decrease was noted in the cinnamates and amino acids clusters. In particular, the GR24 + AMF treatment mostly modulated coumaroylquinic, cinnamic, and coumaric acids, as well as amino acids such as arginine, aspartate, glutamate, glutamine, lysine, and phenylalanine. Other than these, but to a lesser extent, alkaloids, glucosinolates, and lignans were positively affected by the fungi and strigolactones combination application.

### 3.6 Data Integration Analysis

To explore the metabolic responses in tomato roots and root exudates under different treatments, we applied a data integration approach using the DIABLO framework. DIABLO is a supervised multivariate method developed to combine multiple omics datasets into a single model that highlights differences between experimental conditions. In our case, we employed multiblock sparse Partial Least Squares Discriminant Analysis (sPLS‐DA) to identify correlated metabolites that distinguish between AMF, GR24, and GR24 + AMF in roots and root exudates.

**FIGURE 5:**
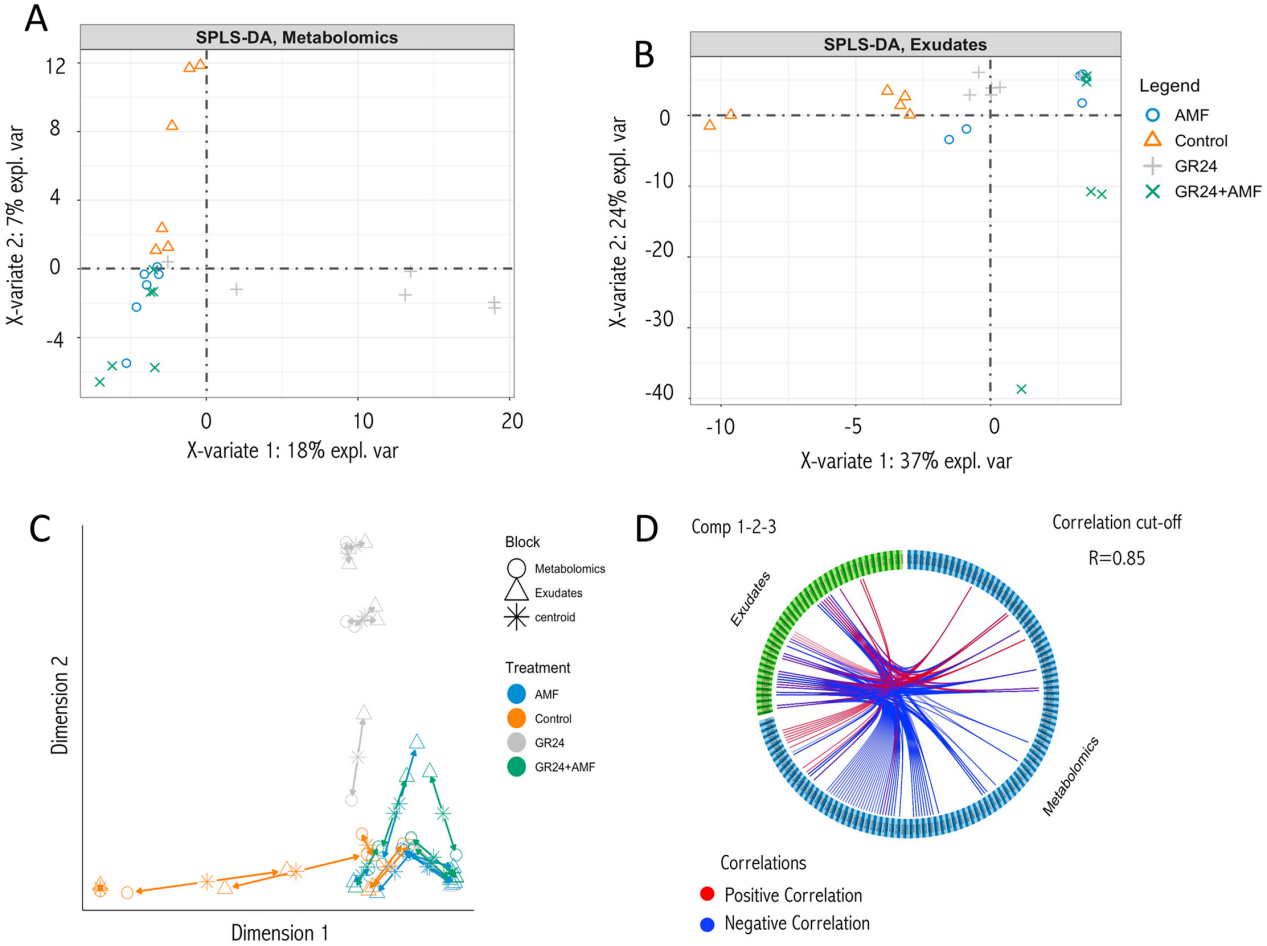
Multi‐omics data integration analysis between tomato root exudates and root metabolic profile treated with 15 mM strigolactone GR24, arbuscular mycorrhiza fungi (AMF), and their combination (GR24 + AMF). (A, B) Sparse Partial Least Squares Discriminant Analysis (sPLS‐DA) model from the DIABLO framework, based on metabolomics profiles of roots and exudates. The samples were plotted on each dataset's first two components and colored according to the treatment. (C) Arrow plot from integrated sPLS‐DA projected into the space. The start of the arrow indicates the centroid between the datasets, and the tip is the location of the same sample in each block. (D) Correlation circle plot between roots and exudates datasets among the features selected from the three considered components (comp 1‐2‐3). The red line represents positive correlations, and the blue line represents negative correlations between datasets. Correlation cut‐off = 0.85.

In the root metabolomics model plot (Figure 5A), GR24‐treated samples were discriminated from the others with positive weights along the first component, indicating distinct metabolic signatures. On the second component, the main separation was instead reported between the controls and the three different treatments considered (Figure 5A). A similar analysis was performed on the root exudate data (Figure 5B), where the selected features pointed out partial discrimination on the first and the second components, mostly between controls and treatments. These results were further supported by the arrow plot (Figure 5C), which visualizes how each sample shifts between the root and exudate datasets. The direction and length of the arrows reflect the degree of similarity or divergence between the two datasets for each sample. Specifically, the controls were distinguishable on the first dimension, and on the second dimension, the differences were mainly between the GR24 and the others.

The circle plot (Figure 5D) identified the most discriminant and correlated metabolites between roots and exudates, with a strong correlation coefficient (*r* = 0.85), indicating consistent biological patterns across datasets.

**FIGURE 6:**
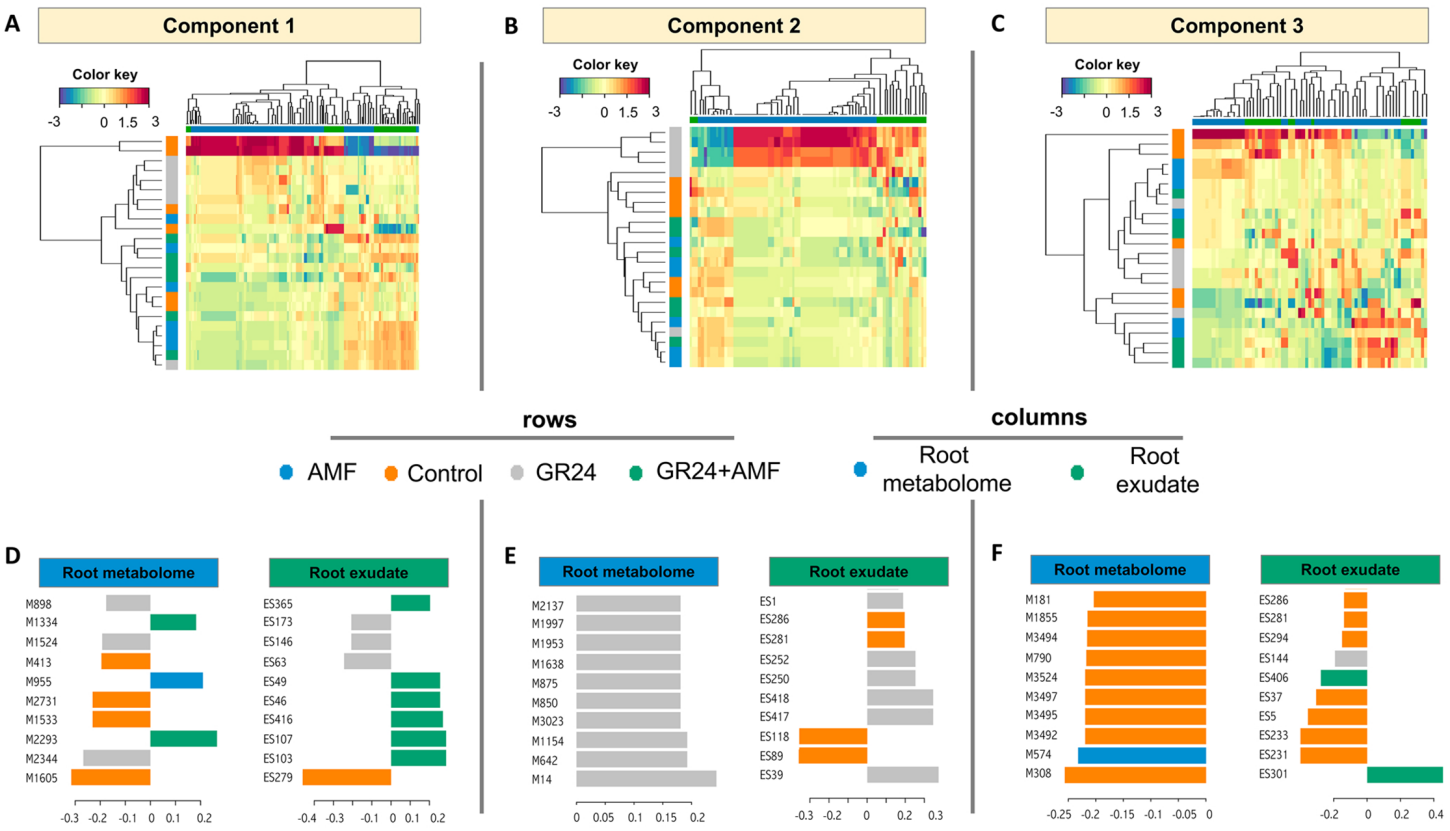
Multi‐omics data integration analysis between tomato root exudates and root metabolic profile treated with 15 mM strigolactone GR24, arbuscular mycorrhiza fungi (AMF), and their combination (GR24 + AMF). (A–C) Heatmaps of the selected features by the sparse Partial Least Squares Discriminant Analysis (sPLS‐DA) across the three components; samples are in rows, and the datasets are in columns. (D) Loading plot of the selected features by sPLS‐DA on component 1, (E) component 2, and (F) component 3. Features are rated according to their loading weights and represented as bars; the colors indicate which class a particular compound is maximally present. The metabolite names are listed in the Supporting Information (Table S3).

After that, the integration of the two datasets was depicted as heat maps (Figure 6A–C), considering the most discriminating compounds of the first three components of the sPLS‐DA model. In Figure 6A,C, two main clusters were generated; one comprising control samples, and another subdivided into AMF and GR24 + AMF treatments, with GR24 samples forming a distinct subgroup. Figure 6B highlights a clear separation between GR24 and the other treatments.

Finally, the loading plots of the 100 selected variables for roots, the 40 from the exudates (sparse component 1; Figure 6D), the 61 and 20 selected variables for sparse component 2 (Figure 6E), and the 51 and 20 selected variables for sparse component 3 (Figure 6F) are presented. These features were selected in accordance with a tuning process that maximizes the correlation between datasets and has high discrimination capacity. All the features recorded an overall Pearson's correlation of *r* = 0.80 for component 1, *r* = 0.91 for component 2 and *r* = 0.66 for component 3. Their loading weights are presented in the Table S4. The features involved in the discrimination pattern of the control and the GR24 with respect to other treatments were mainly represented by metabolites involved in the synthesis of N‐containing compounds (e.g., alkaloids and glucosinolates), anthocyanins, flavones, flavonoids, cinnamates, and phytohormone indole‐3‐acetic acids in roots extracts, and cinnamic acid, kaempferide, and gallic acid in root exudates (Table S4). The GR24 + AMF and AMF treatments were characterized by enhanced synthesis of phytohormones in the root system, mainly represented by gibberellins, cytokinines, and indole‐3‐acetic acids. The hormonal regulation was strictly associated with exudating phosphate acquisition compounds, intermediates for synthesizing fatty acids, and secondary metabolites like flavonoids. Finally, the model reported a clear metabolic profile characterizing the effect of GR24 treatment in tomato roots. The GR24 induced the production and secretion of flavonoids, such as luteolin, hesperidin, quercetin, and derivatives.

## 4 Discussion

Strigolactones are a class of phytohormones that regulate shoot‐root architecture, secondary growth, and leaf senescence whilst acting as stimulants of direct AMF root colonization and hyphal branching (Lanfranco et al. 2018). No differences in plant height were observed between the control group and GR24‐treated plants. Instead, the plants treated with AMF showed better growth parameters compared to those treated only with GR24. Furthermore, no effect on root length was reported following GR24 treatment. A previous study suggested the role of the SL analogue GR24 on root system architecture, reporting a reduction of lateral root density by inhibiting their outgrowth and decreasing their formation potential after GR24 application (Ruyter‐Spira et al. 2011). Generally, in our study, GR24 was applied to the foliage at a concentration of 15 μM. Previous research has demonstrated that GR24 exerts concentration‐dependent effects on plant growth and physiology. For instance, Wani et al. (2023) reported that lower concentrations of GR24 (e.g., 4 μM) significantly enhanced plant height, root length, and biomass accumulation, whereas higher concentrations (e.g., 8 μM) led to a slight decline in these traits (Wani et al. 2023). Similarly, Ahsan et al. (2022) showed that foliar application of GR24 at varying doses (0, 0.001, 0.01, and 0.1 mg l^−1^) resulted in differential responses in shoot and root biomass (Ahsan et al. 2022). These findings suggest that GR24 efficacy is highly dose‐sensitive, and the 15 μM concentration used in our experiment may not have been optimal when applied alone. However, in combination with AMF, GR24 appears to synergize with the fungal symbiosis, enhancing plant performance across multiple parameters.

Strigolactones are known to stimulate the pre‐symbiotic growth of AMF at very low concentrations. In our study, we did not report an increase in AMF colonization under GR24 + AMF treatment compared to AMF applied alone. A plausible hypothesis is that the endogenous SL levels in tomato plants were sufficient to support optimal mycorrhization, as indicated by the comparable root colonization observed between AMF‐only and GR24 + AMF treatments (~25%). This suggests that the exogenous application of GR24 did not further enhance colonization beyond the baseline established by the plant's own strigolactone production. Similar findings were reported by Gomez‐Roldan et al. (2007) in maize, where the application of additional GR24 did not significantly increase AMF colonization (Gomez‐Roldan et al. 2007).

Interestingly, we observed a general enhancement of root FW and DW biomass, compared to other treatments. Generally, the root colonization by AMF is often associated with improvements in root architecture; the relationship is not always linear or directly proportional. In our study, root morphological parameters, including length, volume, surface area, and projected area, were significantly enhanced following AMF and GR24 + AMF treatments compared to non‐treated controls. Although no statistically significant difference was reported between the two, a slight positive tendency was observed in the combination, indicating better performance in root development. Although these changes coincided with a ~25% increase in root colonization relative to controls, it is important to note that the functional impact of AMF depends not only on the extent of colonization but also on the spatial distribution and activity of the fungal structures within the root. Moreover, plants with coarse root systems, characterized by thicker roots and fewer root hairs, tend to benefit more from AMF colonization due to their limited intrinsic nutrient absorption capacity. In contrast, plants with fine, highly branched roots may exhibit less pronounced morphological changes despite similar colonization levels (Maherali 2014). Therefore, the observed tendency for enhanced root architecture without a corresponding increase in colonization suggests that AMF may have exerted a functional influence on root development that is not solely dependent on colonization percentage, but rather on qualitative aspects of the symbiosis (Rouphael et al. 2015).

Additionally, an increase in Phi2 (quantum yield of PSII) and a reduction in PhiNO (non‐regulated energy dissipation) were reported in AMF‐only treated plants. It is reported that the inoculation of AMF adjusts the plant photosynthesis and the performance of the PSII by enhancing water use efficiency, stomatal conductance, and the stabilization of the membrane structure (Gupta et al. 2021). Moreover, inoculated plants result in higher carbon assimilation, increased osmolytes accumulation and antioxidant metabolism that, in turn, positively affect photosynthetic efficiency (Torres et al. 2021). Furthermore, Mathur et al. (2018, 2021) proved the role of AMF in enhancing the physiological resilience of maize plants exposed to extreme heat stress, demonstrating improved photosynthetic performance, including increased quantum efficiency of photosystem II, higher density of active reaction centers, enhanced linear electron transport, and elevated net photosynthesis rates (Mathur et al. 2018, 2021). However, non‐statistical significance was noted for GR24 and GR24‐AMF. This could be a reason associated with the metabolic cost of GR24 and AMF interaction and communication. In general, the effect of AMF on enhancing photosynthetic performance can vary depending on plant species, environmental conditions, and experimental design (Liu et al. 2023). Their effects on fluorescence parameters, such as Phi2 and Fv/fm (the maximum quantum efficiency of PSII), can also vary.

Interestingly, the combined application of GR24 and AMF resulted in a synergistic effect, improving the relative chlorophyll content. Mayzlish‐Gati et al. (2010) reported the effect of SLs application as regulators of plant light‐harvesting by inducing genes putatively associated with components of PSI and PSII, precursors of chlorophyll *a* and *b*, and ribulose‐1,5‐bisphosphate carboxylase (rubisco; Mayzlish‐Gati et al. 2010).

Regarding the root exudate profile, the combination of AMF with GR24 resulted in a positive modulation of phenolic compounds like anthocyanins and flavonoids, amino acids, and cinnamates. The increase of these compounds is usually associated with their roles in plant defense. They help reduce root‐knot nematode invasion (Vos et al. 2012) and enhance insect and pathogenic fungi resistance (Louarn et al. 2012; Tanumihardjo et al. 2020). They also influence the rhizosphere microbial community, improving nutrient cycling and mediating plant–plant interactions through allelopathy by acting as toxins or substrates for beneficial microbes (Zwetsloot et al. 2020). Amino acids can stimulate a group of microbes and suppress many others either by serving as a source of nutrients or by suppressing their growth (Hao et al. 2010). Ma et al., following fungi colonization in maize, reported an enhanced exudation of *p*‐hydroxybenzoic, *p*‐coumaric, and caffeic acids and a decreased level of protocatechuic and ferulic acid (Ma et al. 2022). Moreover, Ren et al. stated that AMF colonization regulated phenolic, amino, and organic acid secretion while suppressing the release of common root exudates (Ren et al. 2015).

The effect of AMF and their combination with GR24 application determined the modulation of the secondary metabolism, particularly nitrogen‐containing related compounds, phenylpropanoids and terpenes. These compounds have been associated with biological functions in plants (i.e., growth and photosynthesis), defense activity against both biotic and abiotic stresses, and antioxidants in the scavenging of ROS (Sharma et al. 2019). Among others, flavanols, anthocyanins, lignans and coumarins were modulated in all the conditions considered. The AMF induces important biochemical modification, causing the accumulation of polyphenols, phenolic acids and carotenoids and enhancing antioxidant enzyme activity in roots and shoots of different plant species (Amani Machiani et al. 2022). In addition, these variations in the composition and the abundance of secondary metabolites mediate the interactions with the plants and create symbiosis with the root tissue (Ganugi et al. 2019). In turn, SLs, flavonoids, terpenoids, and hormone regulators are implicated in the molecular AMF‐plants linkage, underlining the essential role of plant secondary metabolites in the development of AMF (García‐Garrido et al. 2009). Thus, the plant releases organic acids, amino acids, and phenolics as carbon sources for the microbes and flavonoids, which have a stimulatory influence on fungal growth (Wang et al. 2012).

In recent years, many works have focused on the contribution of phytohormones in plant–microorganism interactions (Gutjahr 2014; Pozo et al. 2015) and their involvement in the regulation and cooperation during the establishment of AMF symbiosis, from early recognition to the arbuscular formation and degradation. However, at the molecular level, little is known about the mechanisms of hormone coordination to facilitate the establishment of AMF symbiosis and prevent parasitism by AMF (Liao et al. 2018). Interestingly, in our experiments, GR24 + AMF positively impacted the phytohormone profile with a general increase of abscisic acid, auxins, brassinosteroids, and cytokinins, confirming their pivotal role in the modulation of AMF symbiosis. The combined treatment also resulted in increased jasmonates, which were not observed in the other two treatment conditions. Accordingly, depending on plant species and nutritional conditions, jasmonates can affect mycorrhizal colonization, from positive to inhibitory (Foo et al. 2013). Specifically, jasmonate levels tend to rise during the later stages of AMF colonization in roots, where they help regulate fungal proliferation and maintain the interaction within a mutually beneficial range rather than allowing it to shift toward pathogenicity (Nair et al. 2015). In our study, the hormonal patterns observed under the GR24 + AMF treatment are consistent with this regulatory mechanism, suggesting a tightly coordinated hormone crosstalk that supports a balanced symbiosis and contributes to overall improvements in plant performance. Moreover, Herrera‐Medina et al. (2007) highlighted that abscisic acid reduced the frequency and intensity of root colonization with decreased arbuscular morphology and development (Herrera‐Medina et al. 2007). This outcome is consistent with our finding that the AMF‐GR24 treatment strongly increased abscisic acid synthesis.

Interestingly, AMF, and in particular their combination with GR24, induced a selective increase in gibberellins. The role of gibberellins and their involvement in symbiosis is still poorly understood, but it appears that AMF colonization leads to a substantial increase in gibberellin levels in mycorrhizal roots (Ortu et al. 2012; Liao et al. 2018) by improving the nodulation (Ferguson et al. 2011).

Furthermore, specific hormonal regulation was observed for auxin accumulation (induced by AMF) and brassinosteroid accumulation (induced by GR24). This pattern suggests that the physiological response triggered by GR24 is mediated through a level modulation of the local auxin dynamics, which may depend on the auxin status and/or the plant sensitivity. Notably, the activity of brassinosteroids is functionally integrated with that of the SLs, as both phytohormones act as key regulators of root development. Their coordinated signaling modulates root architecture and contributes to the balance between normal growth and adaptive responses to environmental stress (Altamura et al. 2023).

## 5 Conclusions

This study demonstrates that AMF and GR24, especially when combined, induce specific physiological adjustments that enhance tomato growth, root system architecture, and PSII performance. Both AMF and GR24 + AMF significantly increased root length, volume, and surface area, indicating functional improvements in root architecture that were not directly proportional to colonization percentage, which remained similar between the two treatments. At the photosynthetic level, AMF increased Phi2 and reduced PhiNO, confirming an improvement in PSII efficiency. Although GR24 and GR24 + AMF did not show significant differences in these parameters, the combined treatment enhanced relative chlorophyll content, suggesting a synergistic influence on light‐harvesting processes. Metabolomic and exudate analyses further supported these treatment‐specific responses. AMF and GR24 + AMF modulated phenolic compounds, amino acids, and cinnamate derivatives in root exudates, while the combined treatment produced stronger shifts in secondary metabolism, particularly phenylpropanoids, terpenes, and nitrogen‐containing compounds. These changes align with known roles of AMF and SL‐related signaling in regulating defense‐related and antioxidant pathways. Finally, the hormonal profile revealed that GR24 + AMF induced a broader increase in ABA, auxins, brassinosteroids, cytokinins, and jasmonates compared to either treatment alone, indicating a cooperative modulation of the hormonal network involved in symbiosis and root development. Overall, our findings show that while GR24 alone exerts limited morphological effects, it significantly enhances several AMF‐mediated physiological, metabolic, and hormonal responses. These results highlight the combined GR24 + AMF interaction as a key regulator of plant performance and provide a foundation for future studies exploring strigolactone‐mediated processes under nutrient limitations or abiotic stress.

## Author Contributions

Biancamaria Senizza: contributed to data curation, performed formal analyses, and participated in writing the original draft. Leilei Zhang: conceived the study, contributed to data curation, developed the methodology, managed software and validation activities, wrote the original draft, and supervised the research. Begona Miras‐Moreno: conceived the study, conducted formal analyses, contributed to data curation, and led the investigation. Pascual Garcia‐Perez: performed formal analyses, contributed to data curation, and supported the investigation. Paolo Bonini: contributed to data curation and carried out formal analyses. Fabrizio Araniti: contributed to the methodology and supported data curation. Luigi Lucini: conceived the study, contributed to data curation, supervised the research, provided resources, and revised and edited the manuscript.

## Funding

Leilei Zhang received a fellowship from the Doctoral School on the Agro‐Food System (AgriSystem) of the Università Cattolica del Sacro Cuore (Piacenza, Italy). Pascual Garcia‐Perez received the “Margarita Salas” postdoctoral grant supported by the European Union through the “NextGenerationEU” program provided by the University of Vigo. This research received no specific grant from funding agencies in the public, commercial, or not‐for‐profit sectors.

## Conflicts of Interest

The authors declare no conflicts of interest.

## Supporting information


**Figure S1:** Root Arbuscular Mycorrhizal Fungi colonization.
**Figure S2:** Unsupervised hierarchical cluster analysis of root exudates.
**Figure S3:** Principal component analysis of root exudates.
**Table S1:** Root morphology parameters.
**Table S2:** Dataset of root metabolomics.
**Table S3:** Dataset of root exudates.
**Table S4:** Loading features of DIABLO model.

## Data Availability

The data are provided as Supporting Information.
